# Investigation of genotoxic effect of octyl gallate used as an antioxidant food additive in *in vitro* test systems

**DOI:** 10.1093/mutage/gead005

**Published:** 2023-03-07

**Authors:** Ece Avuloglu Yilmaz, Deniz Yuzbasioglu, Fatma Unal

**Affiliations:** Department of Health Information Systems, School of Technical Sciences, Amasya University, Amasya, Turkey; Science Faculty, Department of Biology, Genetic Toxicology Laboratory, Gazi University, Ankara, Turkey; Science Faculty, Department of Biology, Genetic Toxicology Laboratory, Gazi University, Ankara, Turkey

**Keywords:** octly gallate, food additive, genotoxicity, human lymphocytes

## Abstract

Several antioxidant food additives are added to oils, soups, sauces, chewing gum, potato chips, and so on. One of them is octyl gallate. The purpose of this study was to evaluate the potential genotoxicity of octyl gallate in human lymphocytes, using *in vitro* chromosomal abnormalities (CA), sister chromatid exchange (SCE), cytokinesis block micronucleus cytome (CBMN-Cyt), micronucleus-FISH (MN-FISH), and comet tests. Different concentrations (0.031, 0.063, 0.125, 0.25, and 0.50 μg/ml) of octyl gallate were used. A negative (distilled water), a positive (0.20 μg/ml Mitomycin-C), and a solvent control (8.77 μl/ml ethanol) were also applied for each treatment. Octyl gallate did not cause changes in chromosomal abnormalities, micronucleus, nuclear bud (NBUD), and nucleoplasmic bridge (NPB) frequency. Similarly, there was no significant difference in DNA damage (comet assay), percentage of centromere positive and negative cells (MN-FISH test) compared to the solvent control. Moreover, octyl gallate did not affect replication and nuclear division index. On the other hand, it significantly increased the SCE/cell ratio in three highest concentrations compared to solvent control at 24 h treatment. Similarly, at 48 h treatment, the frequency of SCE raised significantly compared to solvent controls at all the concentrations (except 0.031 μg/ml). An important reduction was detected in mitotic index values in the highest concentration at 24 h treatment and almost all concentrations (except 0.031 and 0.063 µg/ml) at 48 h treatment. The results obtained suggest that octyl gallate has no important genotoxicological action on human peripheral lymphocytes at the concentrations applied in this study.

## Introduction

Antioxidants as food additives, are used to delay oxidation, especially in fat and oil-containing foods. As a result of oxidation, spoilage, rancid taste (bitterness due to the release of fatty acids) and aroma, color change and decrease in nutritional value occur in foods. Antioxidants prevent the deterioration and rancidity of food for a certain period by delaying the effect of atmospheric oxygen at normal temperatures during the production, storage, transportation, and marketing of vegetable and animal oils and other foodstuffs containing fat [1–4]. Antioxidants used in the food industry can generally be classified into four groups: forming a complex with free radicals, reducers, chelating agents, and seconder antioxidants [1–3]. Among the antioxidants that form complexes with free radicals are gallic acid esters. Of these, octyl gallate (E 311) is a food additive that can be used alone or in combination with other gallates in fats and oils, as well as flavorings [5].

Many chemicals which either directly or indirectly affect DNA may play a role in genotoxicological processes that may be active in individuals and beyond generations. They can cause many pathological effects such as carcinogenesis and reproductive system problems [6–8]. It is now recognized that genotoxicity caused by exogenous factors is a significant health threat [9].

Genotoxicity assessment involves performing a wide variety of assays that each is sensitive to different types of DNA damage. It is known that no single test can reliably detect the three main types of genetic damage such as gene mutation, structural chromosomal damage, and aneuploidy). Chromosome aberrations (CAs), micronucleus (MN), sister chromatid exchange (SCE), and comet tests are the most frequently used cytogenetic tests in genotoxicity identification analyses [10–12]. CA assay is effective in detecting both the clastogenic and aneugenic effects of the tested substance [13–15]. Chromosome instability revealed by SCE is closely related to the negative effect of a substance influencing the replication mechanism and repair of DNA damage [16–19]. The cytokinesis block micronucleus cytome (CBMN-Cyt) assay allows for the observation of micronucleus (MN), nucleoplasmic bridges (NPBs), and nuclear buds (NBUDs). MNs are indicative of chromosomal breaks and/or complete chromosome loss, NPBs are indicative of incorrect DNA repair and/or telomere end-lesions and NBUDs are indicative of nuclear elimination of amplified DNA and/or DNA repair complexes [20]. On the other hand, comet assay is a sensitive, reliable, and fast method used to examine the rate and repair of DNA damage [21, 22]. This assay enables the detection of DNA single and double-strand breaks in the cell and the observation of DNA damage at the cellular level [23].

Gallic acid esters are used as antioxidant additives in the food industry as well as in the cosmetics and pharmaceutical industries. The protective effect of these compounds against damage caused by reactive oxygen species was demonstrated by some research [24, 25]. Propyl gallate, one of the gallic acid esters, was extensively studied for its toxicological properties. However, due to insufficient toxicological research, the European Regulation (EU, 2018/1481 2018) [26] has banned the use of octyl gallate (E 311) [4]. Some of the previous studies showed that octyl gallate was not genotoxic [2, 27], but a few studies showed that it may have genotoxic potential [28, 29]. Due to conflicting results, this study aimed to investigate the *in vitro* genotoxic potential of octyl gallate using five different genotoxicity tests (chromosome aberrations, sister chromatid exchange, cytokinesis block micronucleus cytome, micronucleus-FISH, and comet tests). Thus, the main goal of this study was to complete the lack of genotoxicological studies related to octyl gallate.

## Material and methods

In this study, the possible genotoxic effects of octyl gallate (Cas No: 1034-01-1), an antioxidant food additive, were investigated. For this purpose, cultured human peripheral lymphocytes for chromosomal aberrations, sister chromatid exchange, cytokinesis block micronucleus cytome, and micronucleus fluorescence *in situ* hybridization assays; isolated lymphocytes were used for the comet assay. Peripheral blood was obtained from two females and one male (aged 23–27 years) donors who do not smoke, use alcohol or medical drugs, have no health problems and have no known exposure to genotoxins. The study was carried out with the permission of Gazi University Clinical Research Ethics Committee 11.06.2018/452.

### Chromosomal aberrations and sister chromatid exchange assays

Chromosomal aberrations assay in human peripheral lymphocytes was performed according to Evans *et al.*’s [30] method with some modifications [31]. Sister chromatid exchange assay was performed according to Perry and Wolff’s [32] method with some changes [33]. Accordingly, 0.2 ml of peripheral blood was inoculated into 2.5 ml chromosome medium (Chromosome medium B) under sterile conditions. 10 µg/ml of BrdU (5-Bromo-2 deoxyuridine = Bromodeoxyuridine) solution was added to this medium. Then, the culture tubes were incubated for 72 h in an incubator at 37°C. Five concentrations of octyl gallate to be studied were determined according to the LD_50_ doses specified in the literature and results of preliminary trials. These are 0.031, 0.063, 0.125, 0.250, and 0.500 µg/ml. Lymphocyte culture was treated with determined concentrations of octyl gallate at 24 and 48 h after the beginning of the culture period. In addition, a negative control (distilled water), a solvent control (8.77 µl/ml methanol), and a positive control (MMC, 0.20 µg/ml) were used. At the 70th h of culture, 0.06 µg/ml of colchicine solution was added to each tube. At 72nd h, the culture tubes were centrifuged at 1200 rpm for 10 min and the supernatant was removed. 0.075M KCl solution was added to the culture tubes and tubes were incubated at 37°C for 30 min, then centrifuged at 1200 rpm for 10 min and the supernatant was discarded. Methanol:acetic acid (3:1) was added to the tubes and the fixation procedure was repeated two more times. The cell suspension at the bottom of the tube, which was homogenized by pipetting, was spread on previously cleaned slides in different areas. Slides were allowed to dry for 24 h at room temperature. To observe SCEs, fluorescent + Giemsa staining was performed in line with the method developed by Speit and Haupter[33]. In order to determine the number of SCEs, 25 cells (75 cells in total for a single dose) were examined in the preparations of each donor, with well-distributed chromosomes and undergoing second mitosis. To calculate the replication index (RI), 100 cells (a total of 300 cells for each dose) were examined from each of the slides prepared from the donors. RI was calculated according to the following formula: RI = [1 × (M1) + 2 × (M2) + 3 × (M3)]/*N* where M1, M2, and M3 represent the number of cells undergoing first, second, and third mitosis, *N* total number of metaphase scored. For CAs, slides were stained with 5% Giemsa, fixed with entellan. To detect chromosomal abnormalities, 100 metaphases with well-spread chromosomes (a total of 300 metaphases for each concentration) were examined for each donor in each treatment group. In the determination of the mitotic index (MI) 1000 cells (a total of 3000 cells for each concentration) were evaluated from each donor. MI was calculated using the standard formula: MI (%) = [number of mitotic cells/total number of cells scored] × 100.

### Cytokinesis block micronucleus cytome and micronucleus fluorescence *in situ* hybridization assays

The methods described by Fenech [20, 34] were used with some modifications [35] for the CBMN-Cyt test. Firstly, 0.2 ml of 1/10 heparinized peripheral blood was added into tubes containing 2.5 ml of chromosome medium (chromosome medium B). These tubes were incubated for 72 h at 37°C. Cells were treated with concentrations of octyl gallate for 48 h. In addition, a negative, a solvent (8.77 µl/ml methanol), and a positive control (MMC, 0.20 µg/ml) were used. To inhibit cytokinesis, 5.2 µg/ml cytochalasin-B was added at the 44th h. At the end of the culture period, the tubes were centrifuged, then the supernatant was discarded. KCl solution was added to the cells, then the tubes were centrifuged again, the supernatant was discarded and a cold fixative consisting of 3:1 methanol:acetic acid was added. After this process was repeated two times, formaldehyde was added to the last fixative and centrifuged for the last time. The suspension was spread by dripping onto previously cleaned slides. They were left at room temperature for 24 h to dry. A total of 3000 binucleated cells (1000 from each donor) were examined in terms of MN, NPBs, and NBUDs. To determine nuclear division index (NDI), a total of 1500 cells were evaluated in each treatment group (500 each from two female and one male donors). NDI was calculated as follows: [1 × (*1N*) + 2 × (2*N*) + 3 × (3 *N*+ 4*N*)]/*n* where *N*1–*N*4 represent the number of cells with 1–4 nuclei, respectively, and *n* is the total number of cells scored. For the micronucleus-FISH method, the slides were treated with 0.4% pepsin prepared in 0.01 M HCl at 37°C for 10 min. Then the slides were washed in SSC solution and treated with 1% formaldehyde. Paracentric probe was added to the preparations washed in SSC again, and the probes were denatured rapidly at 68°C for 8 min. Subsequently, these slides were incubated at 37°C for 16 h. The preparations were stained after washing in SSC-Tween 20 solution. Binucleate cells containing micronuclei were evaluated as centromere-positive and centromere-negative. 400 binucleated cells (1200 total cells for each concentration) were examined from each donor. Evaluation was performed with a DAPI filter on an Olympus BX51 microscope.

### Comet assay

The method of Singh *et al.* [36] was used for the comet assay. Blood from healthy donors was isolated using biocoll. Isolated lymphocytes were carefully collected and incubated for 1 h at 37°C with the determined concentrations of octyl gallate. A negative, a solvent (8.77 µl/ml methanol) and a positive (100 μM, H_2_O_2_) control group were evaluated in the study. When the application period was complete, the lymphocytes were mixed with low melting temperature agar and spread on previously coated slides with high melting temperature agar. After the preparations were incubated in the refrigerator, they were taken into the lysing solution. Afterwards, the preparations were kept in an electrophoresis buffer and then electrophoresis was applied. After electrophoresis, the preparations kept in neutralization buffer were stained with EtBr. In the evaluation of the comet test, a total of 300 cells, 100 from each donor, were examined for each concentration. Examination was performed on a fluorescent microscope using the “Comet Assay IV” (Perceptive Instruments Ltd., UK) analysis system. Observed DNA damage was evaluated in terms of tail length, % tail density and tail moment.

## Results

None of the applied concentrations of octyl gallate induced a significant change in the frequency of chromosomal aberrations in both 24 and 48 h of treatment compared to the solvent control (Table 1). Several structural abnormalities were observed at octyl gallate treatments and these are shown in Table 1. However, there was no statistically significant difference compared to the control.

**Table 1.** Effect of octyl gallate on chromosomal aberrations in human lymphocytes.

Test substance	Treatment		Aberrations						Abnormal cell ± SE (%)	CA/cell ± SE
	Period (h)	Concentrations (μg/mL)	ctb	csb	scu	dic	ex	f		
Control	24	0.00	5	–	3	1	–	–	3.00 ± 0.985	0.030 ± 0.001
Solvent control	24	8.77 µl/ml	3	1	6	2	–	1	4.30 ± 1.171	0.043 ± 0.011
MMC	24	0.20	14	8	12	9	14	1	17.33 ± 2.185	0.193 ± 0.023
Octly gallate	24	0.031	3	3	2	1	–	1	3.30 ± 1.031	0.033 ± 0.010
		0.063	3	2	5	3	–	–	4.30 ± 1.171	0.043 ± 0.011
		0.125	7	1	3	–	1	–	4.00 ± 1.131	0.040 ± 0.011
		0.250	3	2	3	5	–	1	4.70 ± 1.222	0.047 ± 0.012
		0.500	7	5	2	–	–	–	4.70 ± 1.222	0.047 ± 0.012
Control	48	0.00	3	2	3	1	–	1	3.30 ± 1.031	0.033 ± 0.010
Solvent control	48	8.77 µl/ml	3	1	5	3	–	–	4.00 ± 1.131	0.040 ± 0.011
MMC	48	0.20	17	14	9	6	12	4	19.00 ± 2.265	0.207 ± 0.023
Octly gallate	48	0.031	1	1	5	3	–	1	3.70 ± 1.081	0.037 ± 0.011
		0.063	3	1	7	2	–	2	5.00 ± 1.258	0.050 ± 0.013
		0.125	4	4	5	–	1	–	4.70 ± 1.222	0.047 ± 0.012
		0.250	5	2	4	4	–	–	4.70 ± 1.222	0.050 ± 0.013
		0.500	3	2	6	3	–	2	5.30 ± 1.293	0.053 ± 0.013
Frequency of abnormalities (%)			29.10	17.16	31.34	15.68	1.50	5.22		

ctb, chromatid break ; csb, chromosome break; scu, sister chromatid union; dic, dicentric chromosome; ex, chromatid exchange; f, fragment.

The effect of octyl gallate on sister chromatid exchange (SCE), replication index, and mitotic index at 24 and 48 h of treatment is presented in Table 2. Octyl gallate significantly increased SCE formation in the three highest concentrations (0.125, 0.250, and 0.500 µg/ml) in the 24-h treatment period compared to solvent control, and this increase is concentration dependent (*r* = 0.97). In the 48-h treatment, the frequency of SCE showed a significant increase compared to the solvent control at all concentrations except for the lowest concentration, and this increase also depends on the concentration (*r* = 0.84). Minimum number of SCEs was 1 and the highest number of SCEs was 10 (Table 2). The mitotic index decreased significantly only at the highest concentration in the 24-h treatment and at the three highest concentrations in the 48-h. In addition, our studies showed that octyl gallate did not affect the replication index (Table 2).

**Table 2.** Effect of octyl gallate on sister chromatid exchange, replication index and mitotic index in human lymphocytes.

Test substance	Treatment		Min–Max SCE	SCE/cell ± SE	M_1_	M_2_	M_3_	RI ± SE	MI± SE
	Period (h)	Concentrations (μg/ml)							
Control	24	0.00	1–7	2.89 ± 0.17	73	83	144	2.24 ± 0.047	7.30 ± 0.47
Solvent control	24	8.77 µl/ml	1–7	2.92 ± 0.17	95	76	129	2.11 ± 0.050	6.67 ± 0.46
MMC	24	0.20	11–29	18.00 ± 0.45	95	89	116	2.07 ± 0.048	3.43 ± 0.33
Octyl gallate	24	0.031	1–9	3.40 ± 0.18	62	93	145	2.28 ± 0.045	6.33 ± 0.44
		0.063	1–8	3.42 ± 0.19	59	88	153	2.31 ± 0.045	5.90 ± 0.43
		0.125	1–9	4.15 ± 0.21^a^	89	77	134	2.15 ± 0.050	5.70 ± 0.42
		0.250	2–10	4.71 ± 0.23^a^	80	87	133	2.18 ± 0.048	5.63 ± 0.42
		0.500	2–9	4.76 ± 0.24^a^	128	69	103	1.92 ± 0.051	4.83 ± 0.39^c^
Control	48	0.00	1–6	2.57 ± 0.15	98	71	131	2.11 ± 0.050	7.53 ± 0.48
Solvent control	48	8.77 µl/ml	1–6	2.59 ± 0.15	71	81	148	2.26 ± 0.047	7.20 ± 0.47
MMC	48	0.20	7–25	17.23 ± 0.41	112	82	106	1.98 ± 0.050	3.70 ± 0.34
Octyl gallate	48	0.031	1–7	3.05 ± 0.17	57	83	160	2.34 ± 0.045	6.73 ± 0.46
		0.063	1–7	3.41 ± 0.18^a^	84	79	137	2.18 ± 0.048	6.23 ± 0.44
		0.125	1–8	4.16 ± 0.19^a^	74	85	141	2.22 ± 0.047	5.80 ± 0.43^c^
		0.250	1–8	3.87 ± 0.18^a^	84	68	148	2.21 ± 0.050	5.67 ± 0.42^c^
		0.500	1–7	3.77 ± 0.17^a^	98	104	98	2.00 ± 0.047	4.90 ± 0.39^d^

^a^Significantly different from the solvent control *P* < 0.05 (*t* test).

^b^Significantly different from the solvent control *P* < 0.05 (*z* test).

^c^Significantly different from the solvent control *P*< 0.01 (*z* test).

^d^Significantly different from the solvent control *P* < 0.001 (*z* test).

The effect of octyl gallate on the frequency of micronuclei, nucleoplasmic bridges, and nuclear buds at 48 h was shown in Table 3. Octyl gallate did not cause statistically significant changes in MN, NPB, and NBUD frequencies compared to solvent control. In addition, the results showed that octyl gallate did not affect the NDI (Table 3). According to the MN-FISH test results, no significant change was observed in the frequency of centromere-positive (C+) or centromere-negative (C−) MNs compared to the solvent control (Table 4).

**Table 3.** Effect of octyl gallate on micronucleus, nuclear bud, nucleoplasmic bridge, and nuclear division index in human lymphocytes

Test substance	Treatment		Binucleated cells (BN) scored	Distribution of BN cells according to the number of MN			MN ± SE (%)	Nuclear division index (NDI) ± SE	Nuclear Bud (NBUD) ± SE (%)	Nucleoplasmic Bridge (NPB) ± SE (%)
	Period (hour)	Concentrations (μg/ml)								
				(1)	(2)	(3)				
Control	48	0.00	3000	7	1	–	0.30 ± 0.10	1.68 ± 0.33	0.27 ± 0.09	0.17 ± 0.08
MMC	48	0.20	3000	43	2	1	1.67 ± 0.23	1.67 ± 0.33	1.00 ± 0.18	1.03 ± 0.18
Solvent control	48	8.77 µl/ml	3000	9	1	–	0.37 ± 0.11	1.60 ± 0.32	0.27 ± 0.09	0.20 ± 0.08
Octyl gallate	48	0.031	3000	8	–	–	0.27 ± 0.09	1.58 ± 0.32	0.23 ± 0.09	0.27 ± 0.09
		0.063	3000	9	2	–	0.37 ± 0.11	1.58 ± 0.32	0.20 ± 0.08	0.23 ± 0.09
		0.125	3000	8	2	–	0.33 ± 0.10	1.60 ± 0.32	0.33 ± 0.10	0.27 ± 0.09
		0.250	3000	12	–	–	0.40 ± 0.12	1.44 ± 0.31	0.27 ± 0.09	0.30 ± 0.10
		0.500	3000	14	–	–	0.47 ± 0.12	1.39 ± 0.30	0.43 ± 0.12	0.33 ± 0.10

**Table 4.** MN-FISH test results using pan-centromeric DNA probe.

Test substance	Treatment		Binucleated cells (BN) scored	C+	C−	MN total	C+ (%)	C− (%)
	Period (h)	Concentrations (μg/ml)						
Control	48	0.00	1200	3	12	15	20	80
MMC	48	0.20	1200	22	39	61	36.07	63.93
Solvent control	48	8.77 µl/ml	1200	3	11	13	23.08	76.92
Octyl gallate	48	0.031	1200	5	17	22	22.73	77.27
		0.063	1200	6	21	27	22.22	77.78
		0.125	1200	7	17	24	29.17	70.83
		0.250	1200	9	25	34	26.47	73.53
		0.500	1200	7	20	27	25.93	74.07

C+: Centromere-positive, C−: Centromere-negative.

Effect of octyl gallate on comet tail length, tail density, and tail moment for 1-h treatment is given in Table 5. Octyl gallate did not cause statistically significant changes for all three comet parameters compared to solvent control (Table 5).

**Table 5.** Effect of octyl gallate on DNA damage in human lymphocytes.

Test substance	Treatment		Tail intensity (%)	Tail length (μm)	Tail moment
	Period (h)	Concentrations			
Negative control	1	0.0 µg/ml	9.79 ± 0.77	62.29 ± 1.16	2.30 ± 0.24
Positive control (H_2_O_2_)	1	100 μM	21.13 ± 1.20	148.75 ± 6.00	11.70 ± 1.05
Solvent control	1	8.77 µl/ml	9.60 ± 0.91	63.30 ± 1.31	2.91 ± 0.41
Octyl gallate	1	0.031 µg/ml	9.59 ± 0.84	60.32 ± 1.42	2.71 ± 0.31
		0.063 µg/ml	11.78 ± 0.98	65.70 ± 2.14	2.53 ± 0.32
		0.125 µg/ml	8.32 ± 0.83	59.61 ± 1.93	2.12 ± 0.30
		0.250 µg/ml	11.87 ± 0.92	64.10 ± 1.44	2.24 ± 0.15
		0.500 µg/ml	11.35 ± 0.78	65.31 ± 1.06	2.67 ± 0.15

## Discussion

Genotoxic agents that humans are exposed to cause tissue damage, infertility, birth defects, cancer, and some genetic and multifactorial diseases both in the individual and in future generations. It has been determined that there is a strong relationship between genotoxicity and cancer and many compounds that are carcinogens for humans also have genotoxic effects. Serious health problems caused by genotoxic agents have led to the development of short-term genotoxicity tests to detect such agents. Today, these tests can be applied routinely *in vivo* or *in vitro* and there is a consensus about the validity in the world at large. They are also widely used in the determination of the genotoxic and carcinogenic potentials of food additives [37–39].

According to the results obtained from this study, octyl gallate did not cause a significant change in chromosomal abnormality, micronucleus, nucleoplasmic bridge, and nuclear bud frequency in human peripheral lymphocytes. Similarly, octyl gallate application did not make a statistical difference in comet parameters, percentage of centromere positive and negative cells in MN-FISH test compared to solvent control. Moreover, octyl gallate did not affect the replication and NDI values. On the other hand, the SCE/cell ratio increased significantly at all the four highest concentrations (except 0.063 μg/ml at 24 h treatment) compared to solvent control. In fact, the SCE test was removed from the genetic toxicology test guide with the OECD decision (Test no: 479) on 2 April 2014, due to the deficiencies in understanding the molecular mechanism of the effect determined by this test and criticism of its biological significance [40]. However, it is purposed that crosslinking agents are potent SCE inducers, in general, probably as homologous recombination (HR) is necessary to repair the resulting damaged replication forks that occur in the course of crosslink unlocking. HR is an error-free kind of DNA repair. Consequently, SCEs are at present study examined to be more a biomarker of treatment and repair rather than as a mark of mutagenic response as applied in different genotoxic studies [11, 41, 42]. In mitotic index values, a significant decrease was detected in almost all the highest concentrations and application times compared to the control. The mitotic index is considered as a sensitive marker for assessing mitotic changes caused by damage of cell structure or function. The decrease in the mitotic index can be interpreted that the cell stops growing at any time in the interphase, or the cell loses its capacity to reproduce, or the activation of some mechanisms that cause the death of the cell [13, 15]. High concentrations of octyl gallate cause a decrease in the mitotic index, indicating that it may be cytotoxic. However, according to the results obtained from all applied genotoxicity tests, it can be thought that octyl gallate may not pose a significant genetic risk under *in vitro* conditions. Consequently, the results obtained show that octyl gallate did not have a significant genotoxic effect in human lymphocytes at the concentrations applied in this study.

Today, there are safety concerns caused by food additives in society. Even, since some of them have been proven to be toxic, studies on these substances have increased. In the literature review, no study was found on the genotoxic effects of octyl gallate in human lymphocytes. On the other hand, there were only a few studies examining genotoxicity in different cell groups. According to the report published by EFSA in 2015 [27], the SOS chromotest method was performed for octyl gallate in *Escherichia coli* under conditions with and without metabolic activation and no DNA damage was observed. According to the same report, octyl gallate had no available genotoxicity data. When compared to propyl gallate, it was not thought to have a genotoxic effect since it did not carry additional functional groups that can trigger reactivity against DNA or other genotoxic events [27]. In a study conducted in 2017, the genotoxic and antigenotoxic effects of four different alkyl gallates including octyl gallate against H_2_O_2_ were investigated using micronucleus and comet tests in HepG2 cells. In various concentrations of octyl gallate (1, 0.5, and 0.2 µg/ml), neither an increase in micronucleus formation and DNA damage nor a decrease in damages in cells treated with H_2_O_2_ was observed [2]. The results obtained from this study agree with previous studies. In addition, the comet assay was applied in a study conducted in 2005 in which the genotoxicity of gallic acid and its 15 esters (including octyl gallate) were investigated. It was found in Vero cells treated for 90 min that octyl gallate increased DNA damage at concentrations of 100 and 200 µmol/l compared to the control [28]. Another investigation carried out in human peripheral blood mononuclear cells revealed that 1-h pre-incubation of octyl gallate caused a significant increase in DNA migration at all the concentrations tested (0–5 mM) in the comet assay [29]. It is thought that the difference between the results of the study conducted in 2020 and the comet assay results obtained in this study is due to the different cells and concentrations used. Since the number of studies examining the genotoxicity of octyl gallate is limited and the results of these studies are contradictory, and there is no genotoxicity study performed in human lymphocytes, the results of this present study might be filled an important deficiency in the literature.

The antioxidant effects of propyl gallate have been researched well, and it has been reported that it has anti-apoptotic effects and protects the liver. Octyl gallate has also been reported to act on lipids with a mechanism like propyl gallate. On the other hand, gallic acid showed a regulatory effect on normal cell cycle in the human breast cancer cell line [43]. Octyl gallate showed promoted apoptosis in pancreatic ductal adenocarcinoma (PDAC) cells. It increased the level of BNIP3L, a pro-apoptotic protein, and enabled its physical binding to Bcl-2 and Bcl XL. This binding also causes the release of Cytochrome-C from mitochondrial Bax/Bak channels. In the pancreatic ductal adenocarcinoma mouse model, octyl gallate exhibited increased activity against tumor growth, M2-macrophages, and serum levels of HSP90α. In the same study, octyl gallate (10 μg/g/day) was administered to C57BL/6 mice. On the first and second days of this administration, Pnac 02 cells were inoculated into mice to induce tumorigenesis. After 30 days of exposure to octyl gallate, the tumor weight in mice was reduced significantly compared to the control group. Additional *in vivo* experiments showed that 0.500 μg/g bw/day octyl gallate administration significantly inhibited tumor growth [44]. In addition, octyl gallate completely inhibited the growth of the *Streptococcus mutans* biofilm layer and, therefore, it was stated that it can be used as an oral caries preventive agent [45]. Results of many studies have reported that octyl gallate may have possible anticancer, antifungal, antiamyloid, antibacterial, antiviral, and antidiabetic effects [3, 46–53].

Like other various chemicals, food additives must be evaluated for genotoxicity before being put on the market. However, genotoxicity information about some food additives consists of tests that are mandatory for their introduction to the market. Food additives are chemicals that people are constantly exposed to and are widely used today. For this reason, it is crucial to examine the genotoxic effects of these substances by impartial laboratories. The European Food Safety Authority (EFSA) had previously planned to complete the re-evaluation of all food additives authorized for use in the EU prior to 2009. This re-evaluation for octyl gallate (E 311) was published in 2015. EFSA highlighted that a safety concern is unlikely based on usage data for octyl gallate. However, it was stated that an adequate toxicological database in accordance with current guidelines is required for food additive evaluations of octyl gallate [27]. The European Regulation (EU) 2018/1481 2018 [26] also limited the use of octyl gallate due to insufficient toxicological studies. Therefore, it is considered that there is a significant inadequacy in the toxicological risk assessment of octyl gallate and this study may contribute to overcome this inadequacy. In addition, it may also guide the interpretation of the effects of other food additives from the same group.

According to the EFSA (2015) [27] report, there is no data on genotoxicity of octyl gallate and it does not carry functional groups that can induce genotoxic events. The only difference from propyl gallate is an aliphatic chain that it is thought not to cause genotoxicity. Octyl gallate is anticipated to be non-genotoxic and non-carcinogenic *in silico* systems. In addition to all these comments, the antioxidant activity of octyl gallate has been reported by some studies. When the results of this study are added, it can be said that octyl gallate is a food additive that does not have a genotoxic effect in general. However, *in vitro* results need to be supported by *in vivo* results.
